# Pilot Equivalence Study Comparing Different Batches of Topical 0.025% Capsaicin Emulsion: Product Microstructure, Release, and Permeation Evaluation

**DOI:** 10.3390/pharmaceutics13122083

**Published:** 2021-12-04

**Authors:** Francesc Navarro-Pujol, Sanja Bulut, Charlotte Hessman, Kostas Karabelas, Carles Nieto, Francisco Fernandez-Campos

**Affiliations:** 1 Laboratories Reig Jofre, Avda. dels Flors, s/n, Sant Joan Despi, 08970 Barcelona, Spain; fnavarro@reigjofre.com (F.N.-P.); cnieto@reigjofre.com (C.N.); 2 Bioglan AB, Borrgatan 31, 211 24 Malmö, Sweden; sanja.bulut@bioglan.se (S.B.); charlotte.hessman@bioglan.se (C.H.); kostas.karabelas@bioglan.se (K.K.)

**Keywords:** extended pharmaceutical equivalence, equivalence topical products, capsaicin, in vitro release, in vitro permeation, rheology

## Abstract

The European Medical Agency (EMA) has issued a draft guideline on the quality and equivalence of topical products. The equivalence for complex semisolid formulations involves several steps: the same quantitative content, the same microstructure, the same release, and permeation profile. In this paper, several batches of a low strength topical product, which we used as a reference/comparator product, were evaluated according to the recommendations of the EMA draft guideline. The batches were 0.025% capsaicin emulsions from the same manufacturer that were evaluated in terms of droplet size, X-ray diffraction patterns, rheology, release, and permeation profile. The generated data revealed a large batch-to-batch variability, and if the EMA guideline was applied, these batches would not be considered equivalent, although they were produced by the same manufacturer. The result of this work illustrates the difficulties in obtaining equivalence according to the current draft guidelines. It also highlights that the equivalence guidelines should consider the variability of the comparator product, and in our opinion, the guidelines should allow for claiming equivalence by comparing the limits in the variability of the data generated for the comparator product with the limits in the variability of the data generated for the intended equivalence product.

## 1. Introduction

The European Medical Agency (EMA) recently issued a new draft guideline to study the equivalence of topical products [1]. This guideline established a regulatory framework and a scientific rationale for pharmaceutical companies to develop topical generic products. The equivalence of a test product involved, on the one hand, the extended pharmaceutical equivalence, where the quality attributes of the new formulation, should have the same qualitative (Q1), quantitative (Q2) composition, and microstructure (Q3) as the reference product. In addition, the performance of the test product should be the same as of the reference (using an in vitro release test), and for complex topical formulations, equivalence with respect to efficiency should be shown (using permeation kinetics studies, i.e., an in vitro permeation test). The quantitative composition is usually obtained by reverse engineering [2], and the maximum difference between both products must be ± 5%, except for excipients not related to the performance, which could increase to a maximum of ±10%. The microstructure is evaluated by several techniques, i.e., rheological characterization, droplet size analysis, density, pH, polymorphism (if present), etc. Two in vitro additional equivalence tests are required: the characterization of the drug release (IVRT) and permeation profile (IVPT). For the in vitro release test (IVRT) parameters, release constant at a steady-state, and the maximum release amount at 90% confidence intervals (CIs) must be between 90 and 111%. Finally, for the considered complex formulations (such as emulsions or products with permeation enhancers), in the in vitro permeation test (IVPT) 90% CIs must be between 80 and 125% to claim equivalence. In addition, the EMA draft offered useful information regarding semisolid quality aspects, the tape-stripping technique, and advice on how to reduce variability in equivalent studies. The guideline remains in a draft version today, and several concerns have been raised by the scientific community, including pharmaceutical companies.

In 1998, the United States Food and Drug Administration (US-FDA) issued a guideline [3] for testing the bioequivalence of topical products by dermatopharmacokinetics (DPK). With this technique, different tape strippings were taken at different times from the human stratum corneum of volunteers after formulation administration. With an appropriate analytical method for data and non-compartmental modeling, the area under the curve (AUC) for both the test and the reference product was obtained, and the 90% CI was studied to establish bioequivalence. Unfortunately, owing to the discrepant results of different laboratories when using this methodology [4], the guideline was withdrawn in 2002 [5]. Despite several experimental improvements in DPK [6], the US-FDA did not issue the guideline again. Later, the same agency published non-binding specific product recommendations for studying the bioequivalence of topical products, i.e., for acyclovir creams [7], among others [8]. Here, different criteria are required to demonstrate bioequivalence, compared with the DPK method. The test and reference products must be equivalent in Q1, Q2, and Q3. In addition, The IVRT and the IVPT must be comparable. Many experimental aspects and details of this recommendation appeared in the current EMA draft guideline. In addition, acyclovir topical product from different origins has been extensively studied by various researchers [9,10,11,12] and the sensitivity of IVRT and IVPT methods to discriminate small differences in the product composition/manufacturing process has been demonstrated.

Another guideline used to evaluate the equivalence of topical products are the scale-up and post-approval changes: chemistry, manufacturing and controls, in vitro release testing, and in vivo bioequivalence documentation [13]. It is mainly focused on the performance of IVRT experiments on diffusion cells. The curves of released drug amounts versus the square root of time (based on Higuchi’s equation) are used to obtain the release constant. The 90% CIs are calculated based on a non-parametric approach with the limits of 75–133.3%, which are wider compared to the EMA guideline.

Alternative comparison methods are used in the dissolution test, such as f2 parameters, which are not typically used for semisolids, and the multivariant Mahalanobis distance (MD) when the data variability is high [14]. MD can be obtained from each time point of the release curve (model-independent approach) or from the release parameters (model-dependent approach). The distance between formulations should not exceed 10% to claim equivalence between formulations. The problem with the use of multivariate distance is the inversion of the covariance matrix, which is sometimes not able to manage real data [15]. Finally, a generally accepted methodology is the use of Monte Carlo (MC) simulations. This uses the random estimation of data based on empirical data distribution in order to generate enough data to solve the problem of a low amount of data usually available [16]. This usually penalizes the calculation of the CI obtained with traditional methods because CIs are highly dependent on the sample size.

The equivalence of topical products is a difficult issue to solve, as described in the literature [2,8,17,18,19,20], not only regarding the tight limits described in the EMA guidelines but also considering the intrinsic variability of the IVRT and IVPT methods, the latter also linked to the variability of skin donor samples. In addition, critical quality attributes (CQAs) have an important impact on both IVRT and IVPT. Some CQA examples that affect the product performance are droplet size, particles size (in case of suspensions), viscosity and viscoelasticity properties, polymorphs, pH (which affects the ionization state of the drug), and water activity (related with the subsequent evaporation upon application on the skin) [11,21,22]. In fact, CQAs are composition- and process-dependent. Regarding product composition, many topical products are emulsions in which the surfactants and the oil phase play an essential role in the product microstructure. Lipids of natural origin also have variability in their fatty acid compositions, which leads to the rheological variability of the final product. For example, cetyl palmitate, according to the European Pharmacopoeia monograph [23], is a mixture of esters of lauric acid, myristic acid, palmitic acid, and stearic acid, in different proportions depending on the product manufacturer. Cetostearyl alcohol [24] is a mixture of cetyl and stearyl alcohol (minimum content 40%). Cetostearyl alcohol (type A) emulsifying [25] is a mixture of a minimum of 80% cetostearyl alcohol and a minimum of 7% sodium cetostearyl sulfate. The composition variability of the raw material can also increase the variability of reference products, making it very difficult to achieve the equivalence of topical products. Critical process parameters or CPP (i.e., mixing time and speed, temperature and cooling rate, etc.) could also affect product microstructure and performance [10]. Strategies such as quality by design (QbD) and design of experiments (DoE) allow the effect and the impact of CPP on product characteristics to be evaluated in order to select the most appropriate to obtain equivalence between test and reference products [22]. Finally, the drug transformation on the skin after administration is attracting more attention. The evaporation of volatile solvents in the formula, including water, once it is applied on the skin could cause a concentration increase of the drug substance in the skin. This factor could increase the permeation profile [26], or on the other hand, the high concentration could lead to saturation and crystallization of the drug on the skin surface and reduce the drug absorption [27].

In this work, we studied different batches of a 0.025% (*w*/*w*) capsaicin (CAP) emulsion reference product with respect to microstructure, IVRT, and IVPT. Special attention was given to the rheological profile and very low drug content of this emulsion, which increased the possible variability between the different batches. This research illustrates the difficulties in achieving extended pharmaceutical equivalence according to the requirements of the EMA guidelines.

## 2. Materials and Methods

### 2.1. Materials

A total of 0.025 and 0.075% (*w*/*w*) CAP commercialized reference emulsions were obtained from Alcura-Health supplier (Barcelona, Spain). The raw materials CAP (Alps Pharmaceutical Ind. Co., Ltd., Gifu, Japan), sorbitol (Roquette Freres, Lestrem, France), cetyl alcohol (Cognis (part of BASF), Monheim am Rhein, Germany), glyceryl stearate PEG 100 stearate (Croda, Rawcliff Bridge, UK), isopropyl myristate (Croda, Mevisa, Spain), paraffin, white soft (Calumet Penreco, Karns City, PA, USA), and benzyl alcohol (Lanxess DE GmbH, Berlin, Germany) were used to manufacture the non-commercial formulation CAP 0.05% (*w*/*w*) and the placebo emulsion. Bovine serum albumin and gentamicin sulfate (Sigma Aldrich, Barcelona, Spain), and phosphate buffer (PBS) tablets (VWR, Barcelona, Spain), were used for IVPT experiments.

The non-commercial 0.05% emulsion was manufactured as follows: oil phase components were melted at 65–70 °C; the CAP was suspended in a small portion of isopropyl myristate before being added and mixed to the melted oil phase; sorbitol was dissolved in purified water and heated to 65–70 °C; the water phase was transferred to the oil phase and homogenized at approximately 1500 rpm for 8 min; the emulsion was cooled during slow stirring to <35 °C. The placebo was manufactured in the same way but without CAP. 

### 2.2. Methods

#### 2.2.1. Droplet Size Analysis

To determine the droplet size, an optical microscope (Zeiss Axiostar plus, Carl Zeiss AB, Stockholm, Sweden) with an integrated camera (Canon PowerShot G9, Canon, Solna, Sweden) was used. A small amount of CAP emulsion and placebo emulsion was placed between a coverslip and the glass slide. The pure API was suspended in isopropyl myristate and was also observed under a microscope directly, without the coverslip. Pictures at several fields were observed under polarized and non-polarized light.

#### 2.2.2. X-ray Diffraction Analysis

To examine the crystalline structure of the emulsion, X-ray diffraction was used. The emulsion was placed in a holder with a shallow well (approximately 2 mm). Pure API was mounted on a flat zero-background holder. Samples were scanned in the 3–50° 2theta range, with a step of 0.9° 2theta and a step time of 45 s (56 min in total time), using a STOE-STADI MP instrument with a CuKα1 monochromator and a Mythen1K detector (Stoe-Stadi, Darmstadt, Germany). X-rays were generated at 40 kV and 40 mA.

#### 2.2.3. Rheological Characterization

To determine the rheological behavior, a rheometer (DHR-2, TA instruments, New Castle, DE, USA) was used with a cone-plate geometry of 40 mm, a 53 µm gap for the measurements of the flow curve, and a 500 µm gap for an oscillatory test. A solvent trap cover was employed to minimize the drying of the sample at the exposed edges. All testing was performed in duplicate at 25 °C to measure the viscosity shear rate loop. Shear rate ramped up from 0 to 300 s^−1^ over 60 sec, was held at 300 s^−1^ for 60 s, and ramped down from 300 to 0 s^−1^ over 60 s. An oscillatory stress sweep ranging from 0.1 to 1000 Pa, 1 Hz oscillation frequency, was employed to examine the viscoelastic properties. The rheological parameters studied were viscosity (at a shear rate of 300 s^−1^), relative thixotropic loop area or RTLA (Equation (1)), yield stress, complex modulus (G*), and phase angle.

(1) $$RTLA=\frac{S_{thix}}{S_{asc}}\;\cdot 100$$

*S_thix_*, being the thixotropic loop area and *S_asc_* the area enclosed under the up ramp rheogram.

#### 2.2.4. In Vitro Release Test (IVRT)

An IVRT study was conducted in vertical diffusion Franz cells with a receptor volume capacity of approximately 12 mL and a diffusional area of 1.54 cm^2^. The formulation was dosed in the donor compartment at 600 mg, which was separated from the receptor compartment with a polyethersulfone 0.45 µm membrane. The receptor solution was a solution of ethanol:purified water (50:50) that allowed the maintenance of sink conditions throughout the experiment, with a stirring speed of 500 rpm. All experiments were conducted at 37 °C. Samples of 300 µL were taken at different time points, up to 6 h (0.5, 1, 2, 3, 4, 5, and 6 h) and replenished with a tempered fresh receptor medium. Samples were analyzed with the validated HPLC method described in Section 2.2.7.

IVRT method validation: the linearity of the drug release rate and the maximum released amount at three drug strengths (0.025, 0.05, and 0.075%) was studied (*n* = 12 for each dose strength). The discriminative power of the method was evaluated (*n* = 12) by testing a formulation with 0.025% of capsaicin and a cetyl alcohol concentration of 4% (instead of 8%), which resulted in a reduction of formulation viscosity. To check the method precision, the IVRT study was conducted on two different days by two different operators in triplicate (intermediate precision study, *n* = 12). Finally, to study the formulation robustness, two different levels, upper and lower with regard to the parameters selected, were studied under the conditions described in Table 1. For each parameter level, three replicates were tested.

Equivalence test: three different commercial batches of 0.025% capsaicin were studied based on the previously selected experimental parameters. Twelve replicates were assayed for each commercial batch.

#### 2.2.5. In Vitro Permeation Test (IVPT)

Frozen dermatomed (500 µm) abdominal human skin (Biopredict International, Saint Grégoire, France), obtained after plastic surgery, was used for the IVPT experiments. Written informed consent was obtained from skin donors. Before carrying out the experiment, the skin was thawed and placed in Franz cells, with an effective diffusion surface of 1 cm^2^. The receptor medium was 4% albumin in PBS pH 7.4 and 0.01% gentamicin (at 32 °C), which maintained the skin conditions throughout the experiment. Transepithelial electrical resistance (TEER, Asturias, Spain) was measured before the experiment to check the skin integrity, with a cut-off value of 2000 Ω above values indicating the tissue suitability. Samples were taken at the following time points: 0, 1.5, 2.5, 3.5, 5.5, 7.5, 9.5, 11.5, 14.5, 17.5, 20.5, and 24 h. All samples were analyzed with the HPLC-MSMS method described in Section 2.2.7.

Prior to the equivalence study, a validation of the IVPT method was carried out to check its suitability based on the guideline requirements. The linearity between different product strengths 0.025%, 0.05%, and 0.075% CAP was carried out with a donor compartment emulsion dose of 15 mg/cm^2^ and 50 mg/cm^2^.

Based on the suitability study, a pilot study was carried out. For that purpose, six different skin donors and two replicates per donor were used. A negative control (0.05% capsaicin formulation, used in the IVRT experiments) was used to check method discrimination ability. Two reference batches of 0.025% capsaicin were tested. The formulation was dosed in the donor compartment at 15 and 50 mg/cm^2^. At the end of the experiment, the non-permeated emulsion was removed, and the skin was cleaned with a receptor solution. The skin was subsequently extracted with 10 mL of acetonitrile: 0.1% formic acid water (80:20 *v*/*v*). These samples were analyzed with the same HPLC-MSMS method described in Section 2.2.7 with the appropriate dilutions to meet the method linearity. CAP mass balance was performed using the sum of the drug amounts found in the receptor compartment fluid (after the last experimental data point), the drug amount in the residual emulsion (non-permeated fraction), and the drug retained in the skin samples at the end of the experiment.

#### 2.2.6. Statistical Evaluation

To perform the equivalence test of quantitative physicochemical parameters, the 90% CI of the differences of means between batches was carried out, according to Equation (2). The maximum and minimum CI limits should fall into the 90–110% range.

The release rate at the steady-state of the IVRT experiments (obtained by linear regression of the drug release amount per square centimeter versus square root of time) and the maximum released amount was obtained, and the 90% confidence interval (CI) was calculated (Minitab 17 Statistical Software) according to Equation (3). The CI of the formulation employed in the discrimination power during the validation step should be outside the 90–111% range, and the formulations used in the equivalence test should be within these values. For the evaluation intermediate precision, the relative standard deviation (CV%) was obtained and should be less than 10%. ANOVA was used to evaluate method robustness.

In the IVPT experiments, the cumulative drug concentration per square centimeter versus time was obtained. Linear regression between both parameters was used to obtain the steady-state flux. The geometric mean was obtained from each donor replicate. A 90% CI of the ratio of log-transformed means between different batches was calculated with Phoenix WiNonlin (Centara L.P. Pharsight, St. Louis, MO, USA) for the transdermal flux and the maximum permeated amount at the end of the experiment, according to Equation (3). To claim equivalence, the CI should be within the 80–125% limit. In addition, the CV% of both parameters was obtained.

(2) $$90\%\;CI=\left({\bar{X}}_{test}-{\bar{X}}_{ref}\right)\;\pm t_{1-\frac{\alpha }{2},\;\;\;df}\sqrt{\frac{\sigma_{test}^{2}}{n_{test}}+\frac{\sigma_{ref}^{2}}{n_{ref}}},$$

(3) $$90\%\;CI=\frac{{\bar{X}}_{test}}{{\bar{X}}_{ref}}\;\pm t_{1-\frac{\alpha }{2},\;\;\;df}\sqrt{\frac{\sigma_{test}^{2}}{n_{test}}+\frac{\sigma_{ref}^{2}}{n_{ref}}}.$$

where $\bar{X}$ is the mean value to evaluate the test or reference product (i.e., the release constant, the transdermal flux), *t*_1-*α*/2_ is the Student’s *t* value for *α* = 0.90, df is the degree of freedom, *σ*^2^ is the variance, and *n* is the number of observations.

#### 2.2.7. Analytical Methods for Drug Quantification

For the IVRT drug analysis, an HPLC method was employed. Briefly, an HPLC coupled with a UV detector (Water Corporation, Milford, MA, USA) was used to detect CAP. The mobile phase, composed of water:acetonitrile (60:40), flowed at 1 mL/min through a C18 column (150 × 4.6 mm, 5 µm) and was stored at 30 °C. The wavelength was 215 nm, and the injection volume was 50 µL. The analytical method was validated according to the ICH Q2 validation of analytical procedures [28]. The method was found linear between 0.16 and 41.8 µg/mL, and the LOQ was established at 0.16 µg/mL. Precision and accuracy were demonstrated at 0.16, 11.5, and 34.5 µg/mL.

For the IVPT drug analysis, an HPLC-MSMS method was employed. Briefly, an Agilent series 1100 liquid chromatography system (Agilent Technologies, Santa Clara, CA, USA) was coupled to a mass spectrometer API4000 with a TurboIonSpray ion source (AB Sciex LLC, Framingham, MA, USA). A mobile phase (A: water 0.1% formic acid, B: methanol/acetonitrile 20/80 with 0.1% formic acid) flowed at 0.5 mL/min through a C18 column (50 × 0.3 mm, 5 µm) in a gradient elution (A:B mobile phase composition; 0 min 50:50; 2 min 15:85; 3.5 min 50:50). An amount of 10 µL was injected. A positive multireaction monitoring (MRM) mode was used to detect CAP (Q1 mass 306.3 Da and Q3 mass 136.9 Da) and the internal standard [^13^C, ^2^H_3_]-CAP. The method was validated according to the guidelines of bioanalytical method validation [29]. The method was found linear between 100 and 0.5 ng/mL (LOQ). Precision and accuracy were demonstrated at 1.5, 7.5, 50, and 80 ng/mL.

## 3. Results and Discussion

### 3.1. Physicochemical Characterization of Capsaicin 0.025% Reference Product

The physical characterization of capsaicin 0.025% emulsion was performed using an analysis of the droplet size under light and polarized microcopy.

**Figure 1:**
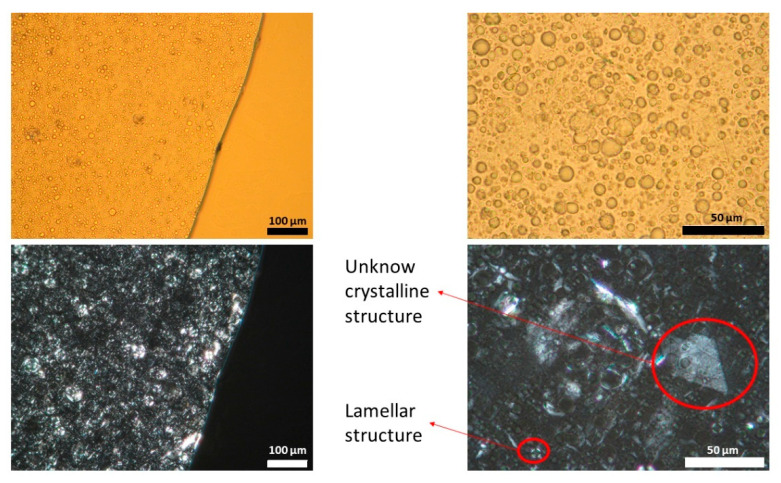
Light (upper panel) and polarized (lower panel) microscope images of batch one capsaicin emulsion at different magnifications. Unknown crystalline structure and lamellar structure is highlighted. A high variability in droplet size could be observed.

**Figure 2:**
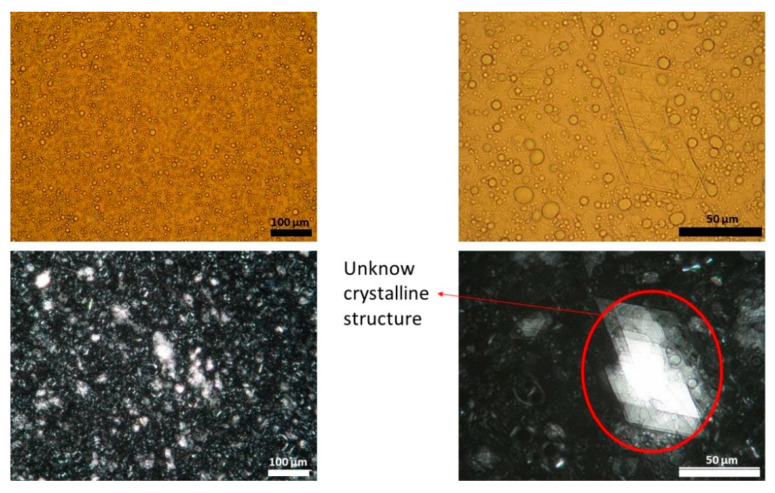
Light (upper panel) and polarized (lower panel) microscope images of batch two capsaicin emulsion at different magnifications. Unknown crystalline structure is highlighted. A high variability in droplet size could be observed.

**Figure 3:**
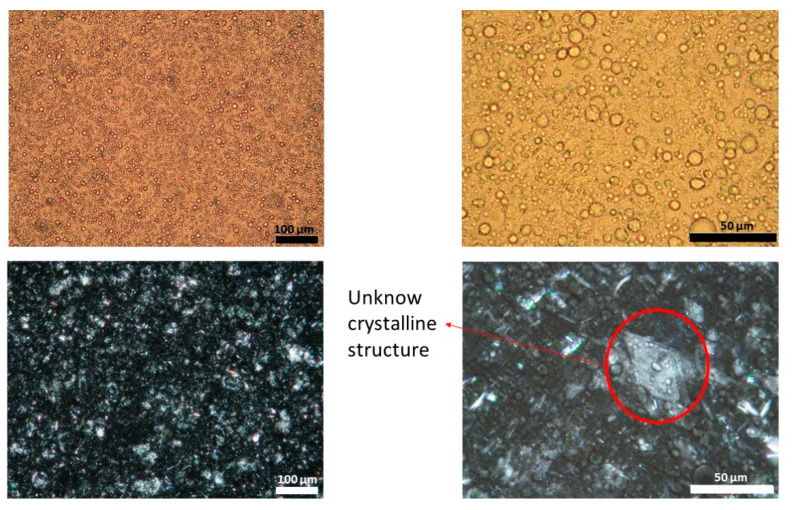
Light (upper panel) and polarized (lower panel) microscope images of batch three capsaicin emulsion at different magnifications. Unknown crystalline structure is highlighted. A high variability in droplet size could be observed.

**Figure 4:**
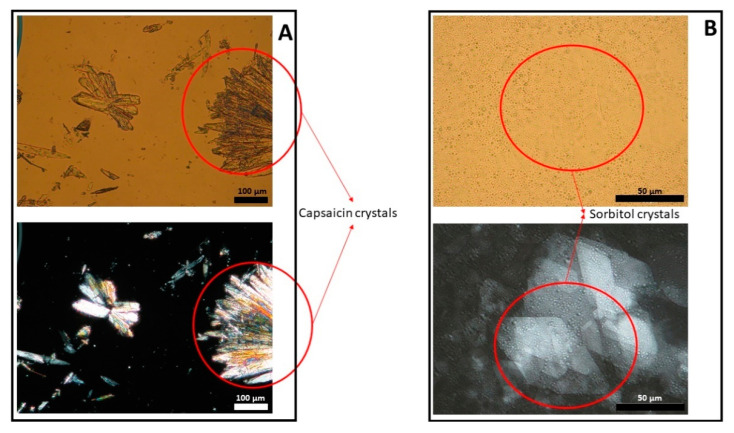
Light (upper panel) and polarized (lower panel) microscope images of capsaicin suspended in isopropyl myristate (panel **A**) and a placebo emulsion (panel **B**).

Figure 1, Figure 2 and Figure 3 show representative images of three different batches of the reference product under light and polarized microscope at different magnifications. As can be seen, there was a heterogenous distribution of the oil droplets. Polarized light allowed us to see the crystalline structure of the emulsion. The typical lamellar structures of surfactant could be observed around the oil droplet. In addition, we observed large crystals that could be another lipid birefringent structure or the non-solubilized API. To ascertain what component was responsible for this crystalline structure, different formulations were prepared: a suspension of CAP in isopropyl myristate (Figure 4A) and a placebo emulsion (Figure 4B). The crystalline structure of CAP in isopropyl myristate was not similar to the objects observed in Figure 1, Figure 2 and Figure 3, but it appeared in the placebo emulsion. Considering the raw material of the emulsion, sorbitol could be supplied as a saturated sorbitol solution (70%) [30]. Even though it might have been supplied as a solution, the different process parameters and the changes in product temperature throughout the emulsification process could be the reason for the crystallization of this excipient in the emulsion.

**Figure 5:**
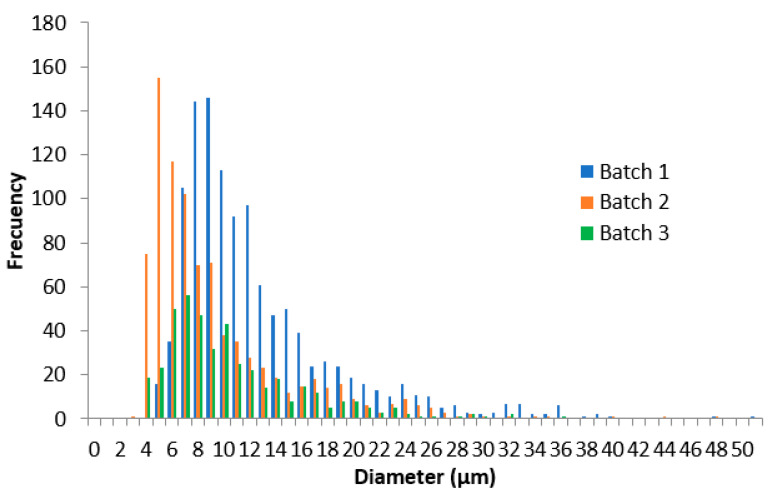
Droplet size distribution of the 0.025% *w*/*w* capsaicin emulsion of three different batches.

The analyses of the droplet size and distribution among the three reference product batches tested were carried out with ImageJ software. The mean droplet size of batch one was 12.16 ± 6.31 µm, 8.86 ± 5.78 µm for batch two, and 10.05 ± 5.29 µm for batch three. The three batches followed a non-normal distribution (Anderson–Darling test, *p* < 0.05) in their droplet size (Figure 5). According to the EMA guideline, to compare physicochemical parameters, a normal distribution is recommended to be assumed, but as the data showed, this premise was not achieved, revealing the guideline inconsistency with empirical data. Nevertheless, following the indications of the guideline, the 90% CIs were obtained, being 1.34–1.40, batch one versus two, 0.86–0.91, batch two versus three, and 1.18–1.24, batch one versus three. These values were outside the limits stated in the guideline by ±10%.

**Figure 6:**
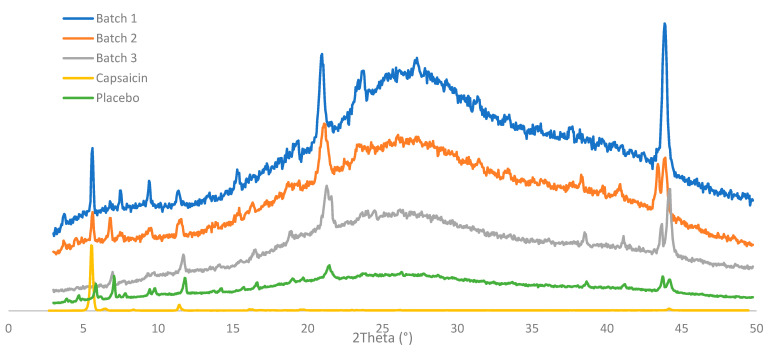
X-ray diffraction patterns of three batches of 0.025% emulsion, pure capsaicin powder, and placebo emulsion.

To undertake a deeper characterization of the crystalline structure, the X-ray diffraction profile was obtained from the three batches of the CAP emulsion, a placebo sample, and the pure API (Figure 6). All samples showed a few sharp reflections in their diffractograms, indicating the presence of crystalline material. However, the background of the X-ray diffractograms for the emulsion samples had the shape of a wide hump, suggesting that the product included a large degree of amorphous components, as expected for a semi-solid product. Two strong peaks were observed at 21.2 and 44.1° 2theta. These peaks were related to each other (harmonics of the same reflection), which suggested the presence of a phase with a repeating distance of approximately 4 Å, potentially a lamellar phase. Data observed in the reference CAP emulsions were deconvoluted into two main types (type I and type II), which shared some diffraction peaks. The characteristic peaks of type I were 3.9, 5.8, 7.8, 9.7, 13.6, 15.6, 19.6, and 23.6° 2theta (±0.2° 2theta), and type II were 4.7, 7.0, 9.4, 14.2, 16.5, 19.0, 33.6, and 41.1° 2theta (±0.2° 2theta). Batch one had only type I reflections, while batches two and three included reflections from both types I and II. It was also noted that the two main reflections from the API (5.9 and 11.7° 2theta) were almost perfectly overlapped with strong reflections of the placebo diffractogram. Thus, it was difficult to detect whether the API was present in crystalline form at the current low concentrations in the emulsion. The variation in diffractogram appearances was an indication of some kind of inhomogeneity between batches.

Finally, a rheological characterization (viscosity at 300 s^−1^, relative thixotropic loop area or RTLA, yield stress, G* or complex modulus, phase angle δ) was carried out. Four different replicates of each CAP emulsion batch at two different time points (around 10 months’ time gap) were performed (Table 2).

As could be seen, the data of intra-batch and intra-time had low variability, but when the two time points were compared in the same batch, the variability increased dramatically. The emulsion underwent an aging process, causing the rheological parameters to drastically modify with time. There was a reduction in viscosity, yield stress, and complex modulus; on the other hand, the relative thixotropic loops area increased slightly, and the phase angle remained more or less constant with time (thus, the relationship between G’ and G” was modified in the same way at different time points). This aging process increased the variability of rheological parameters to a great extent. Even if the same time point (one or two) was considered, the variability of the rheological parameters among batches was still high. When the 90% CIs were compared between batches, the limits were exceeded by ±10%, according to the EMA guideline for all parameters except the delta tangent (Table 3). These results were in line with previous work [19]. Delta value is a relative parameter that balances the elastic and the solid material behavior. Although it exceeded the limits by ±10%, the relative thixotropic loop area had low variability. It is possible that the relative parameters were more adequate to evaluate the formulation sameness, considering the high variability of semi-solid formulations.

### 3.2. IVRT Validation

**Figure 7:**
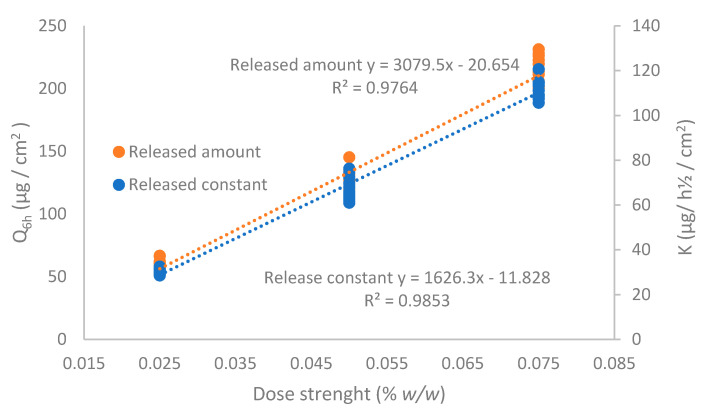
Linearity of the IVRT method for the released amount of capsaicin at 6 h (Q_6h_) and release constant (K).

To evaluate the linearity of the IVRT method, three different CAP emulsion formulations were evaluated at 0.025%, 0.05%, and 0.075% strength according to the method described in Section 2.2.4. The release constant (K) and the maximum released amount at the end of the experiment (Q_6h_) of individual experiments (*n* = 12, each formulation) were obtained, and a linear regression versus drug concentration was performed (Figure 7). As observed, the regression coefficient of both parameters was higher than 0.90, concluding that the method is linear among the tested strengths.

Table 4 show the intermediate precision of the IVRT method. Twelve replicates per operator were carried out, and K and Q_6h_ were determined. The global coefficient of variation was lower than the 10% indicated by the EMA guideline.

To evaluate the discriminatory power of the IVRT method, a negative control with a lower viscosity (achieved with a different concentration of cetyl alcohol) was assayed, checking the 90% CI compared with a reference batch (Table 5). We saw that the CIs were out of the 90–111% range; therefore, the discriminatory ability of the method was demonstrated based on the methodology stated in the EMA guideline. It was also seen that the sensitivity of the IVRT depended on the product viscosity.

To finalize the method validation, the robustness was determined (Table 6) by modifying the release parameters: composition of the receptor medium, the temperature, mixing rate, and dose applied in the donor compartment. For each factor, the Q_6h_ and K were determined, and an ANOVA was carried out. Only the temperature was the significant variable that affected both Q_6h_ and K. Slight variations in the receptor medium, mixing rates, and emulsion dose did not affect the release parameters, as expected.

The variability of viscosity with time, owing to the aging process of this specific emulsion, added more difficulties to carrying out the IVRT experiments. It will be important to set the experiments within a shorter time frame to minimize the viscosity modifications of the formulation.

### 3.3. IVRT Equivalence Test

**Figure 8:**
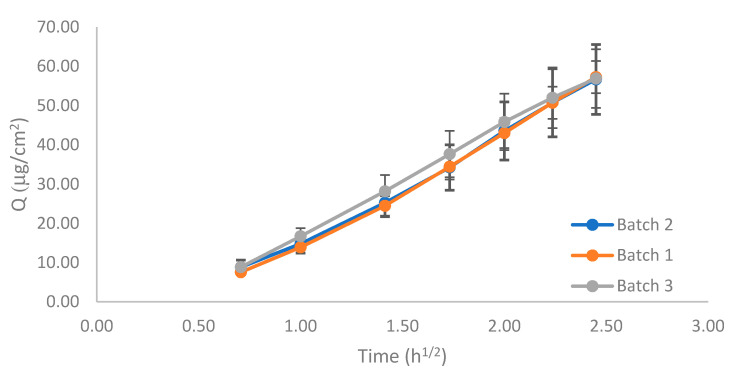
Release profile of the three 0.025% *w/w* capsaicin emulsion batches.

To perform the equivalence test, the three reference emulsions (*n* = 12, each batch) were assayed, according to the standard IVRT method described previously. Figure 8 show the release profiles. The 90% CIs for Q_6h_ and K were calculated to compare the batches (Table 7).

The CI limits of the Q_6h_, or pair comparison, fell within the 90–111% acceptable criteria, but those of K did not. However, there was a large overlap among the release profiles, especially between batches 1 and 2, which was also observed in the K ratios between batches, which were almost 1 in the three comparisons.

### 3.4. IVPT Validation

**Figure 9:**
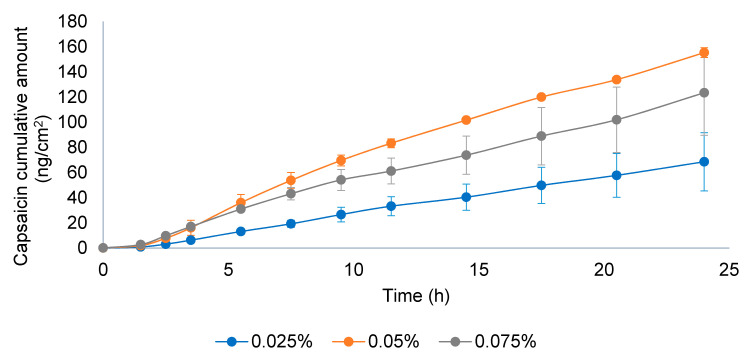
Permeation profile of three capsaicin emulsion batches at 0.025, 0.05, and 0.075% *w*/*w*. Dose in the donor compartment = 15 mg/cm^2^.

**Figure 10:**
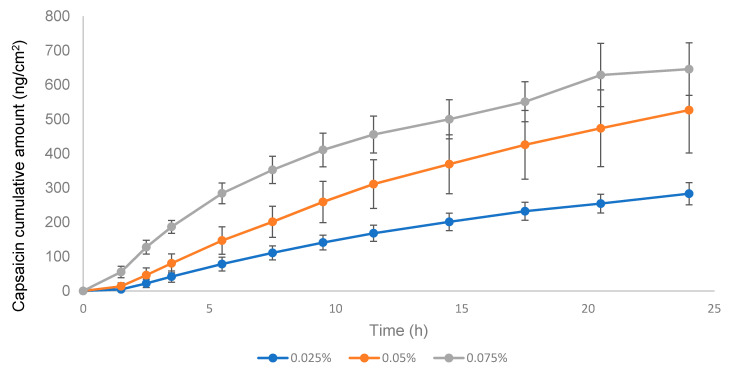
Permeation profile of three capsaicin emulsion batches at 0.025%, 0.05%, and 0.075% *w*/*w*. Dose in the donor compartment = 50 mg/cm^2^.

According to the EMA guideline, the applied dose in the donor compartment should be between 2 and 15 mg/cm^2^; considering the low strength of the product (0.025% *w*/*w*), it was selected as 15 mg/cm^2^ to avoid analytical method sensitivity issues. In the validation study, formulations with CAP concentrations 0.05 and 0.075% were included. The negative control used was the 0.05% w/w formulation used in the IVRT linearity experiments and included the 0.075% w/w to further study the linearity of the IVPT method. As seen in Figure 9, the permeation profile of the 0.05% CAP was higher than the 0.075% CAP, which was not proportional to the API content. The experiment was repeated with an emulsion dose in the donor compartment equivalent to 50 mg/cm^2^ (Figure 10). In this case, logical results were obtained, the transdermal flux and the maximum permeated amount flowed in the order of 0.025% < 0.05 < 0.075%. The lack of linearity found at 15 mg/cm^2^ could be related to the very low dose strength of the product and the low amount of formulation placed in the donor compartment. After observing the drug release from the formulation, the stratum corneum was saturated with the available drug on the skin surface before diffusion to underlying skin layers. The low drug amount available (low strength and low dose in the donor compartment) could obstruct the complete saturation of the stratum corneum and lead to a lack of linearity between doses, obtaining an erratic permeation. With the increase of the emulsion dose in the donor compartment, the stratum corneum could be effectively saturated, and the linearity between doses was achieved. Hence, the conditions stated in the EMA guideline might not be the best ones to follow when the concentration of API in the drug product is very low. Based on these results, the equivalence test was carried out with 50 mg/cm^2^.

### 3.5. IVPT Equivalence Test

**Figure 11:**
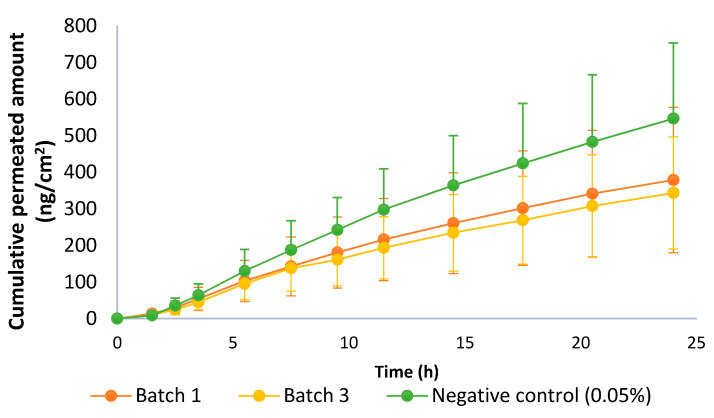
Permeation profile of batches 1 and 3 of the capsaicin emulsion at 0.025% *w*/*w* and negative control (0.05% *w*/*w*). Dose in the donor compartment = 50 mg/cm^2^.

The pilot equivalence test was carried out with batches one and three. Batch three was the most different, compared to batches one and two (which almost overlapped) in the IVRT experiments (Figure 8, IVRT). Thus, batches one and three were selected to challenge the guideline. The negative control concentration was 0.05%, i.e., double the test product. The guideline recommended using a formulation with 50% of the dose strength, but to avoid sensitivity problems, it was decided to increase the drug content. Approximately, 150% of the dose strengths corresponded to 0.0356% but considering the low concentration of the API in the drug product and the variability of the IVPT test, it was considered more appropriate to use 0.05%, which was also used in the IVRT validation study. Results are shown in Figure 11, and the CIs between different batches are shown in Table 8.

Batches one and three exceeded the 80–125% limit, so both batches were considered non-equivalent. Regarding the CI of the negative control versus batch one, which was the closest to the negative control (thus a better case), it did not fulfill the acceptance criteria because the CI must be entirely outside of the 80–125% range, and in this case, the upper limit was within this interval. Thus, there was no discriminatory power.

Finally, the mass balance of the IVPT study was performed, considering the maximum cumulative amount found in the receptor compartment and the non-permeated drug (the drug retained in the skin and the emulsion on the skin at the end of the experiment). In all cases, the overall mass balance was within the range of 90–110%, but there were individual values outside this range. The maximum value is 127.39%, and the lowest value is 79.84%. The guideline only included the requirement for the medium value, but the variability (maximum and minimum values) was not negligible.

As a general discussion, we found several issues when the EMA guideline was applied to semisolid products. We found that different batches of the reference product did not fulfill the requirements for equivalence. For example, the guideline recommended to assume a normal distribution of data (droplet size and other quantitative quality parameters), while the empirical data, as shown here, did not follow this distribution. There was a lack of information about the comparison of qualitative attributes, for example, the comparison of the X-ray diffraction patterns. In this case, multivariate analysis could be applied, but only when the profiles were similar. The CI requirements according to the EMA draft, and the suggested methods to test equivalence, were considered tight, based on the variability of the reference product (as shown in Section 3.1, Section 3.3 and Section 3.5). This variability could be the result of several facts, as described in the introduction section, for example, when considering formulations containing the use of raw materials of natural origin or process-related variability, i.e., non-identical equipment that can show high variability. Regarding low strength products, an additional concern was observed in IVPT experiments that could be related to the low CAP concentration in the emulsion (0.025%) and the low dose administrated in the donor compartment of the IVPT study. The doses used in the IVPT experiments, especially those with low strength products, could also be a major concern. Finally, the CI experimental values were highly dependent on the sample size. The draft guideline proposed the use of twelve skin donors. However, if the CI limits are not achieved, the sample size could be increased based on the statistical calculation (according to bioequivalence studies). This causes a high increase in cost and time when carrying out the experiment for dossier submission. Other approaches could be considered in this case, such as Monte Carlo simulations, to avoid increasing sample size and save time and cost. If it is not possible to obtain equivalence with the proposed draft guideline, the pharmaceutical companies should demonstrate equivalence based on clinical endpoints, which could be even more expensive and time-consuming. Finally, when the reference product has a low rate on the market, i.e., is not produced very often, this fact can increase the time required to carry out the experiments, as the coexistence of several batches on the market with approximately the same shelf-life can be difficult to evaluate regarding the rheology profile, especially products in which the viscosity has changed over time. Therefore, we think that consideration should be given to the variability of the reference product, and the CI limits should be adapted to its variability limits. If equivalence cannot be shown between different batches of the reference product, as illustrated herein, it will be almost impossible to obtain equivalence between the test and reference products.

## 4. Conclusions

An extended pharmaceutical equivalence study was carried out for a capsaicin reference emulsion with several batches of the same product and manufacturer. Several difficulties and/or inconsistencies were found between the experimental parameters and the requirements of the EMA guideline draft. The EMA guideline is still in draft version, and it is expected that some aspects will be adjusted in the future before the final version is issued. These adjustments should include a consideration of the reference product variability when claiming equivalence between a test and a reference product.

## Figures and Tables

**Table 1:** Operational parameters of the IVRT study robustness.

Conditions	Placed Amount of the Product	Mixing Rate of Rec. Medium	Temperature of Receptor Medium	Composition of Receptor Medium
Standard conditions	600 mg	500 rpm	37 °C	Water:ethanol (50:50)
Mixing rate	600 mg	450 rpm	37 °C	Water:ethanol (50:50)
Placed amount of product	630 mg	500 rpm	37 °C	Water:ethanol (50:50)
Temperature of receptor medium	600 mg	500 rpm	32 °C	Water:ethanol (50:50)
Receptor medium compossition	600 mg	500 rpm	37 °C	Water:ethanol (55:45)

**Table 2:** Rheological profile of three batches 0.025% *w*/*w* capsaicin emulsions. Table shows mean value, and values in brackets show the relative standard deviation (CV%). η: viscosity. RTLA: relative thixotropic loop area, G*: complex modulus. δ: phase angle of delta tangent.

Batch	Time	η 300 s^−1^ (Pa)	RTLA	Yield Stress (Pa)	G* (Pa)	δ (°)
1	1	0.354 (1.21%)	0.526 (0.51%)	74.2 (6.35%)	1421 (11.02%)	25.0 (3.87%)
	2	0.276 (2.09%)	0.538 (2.71%)	33.15 (8.02%)	538.8 (10.39%)	28.5 (4.04%)
Intra-batch CV		0.314 (13.73%)	0.532 (2.18%)	53.7 (41.39%)	979.9 (49.39%)	26.8 (7.90%)
2	1	0.347 (1.13%)	0.533 (1.50%)	105 (7.69%)	1620.5 (3.17%)	25.6 (1.29%)
	2	0.210 (5.40%)	0.628 (0.54%)	62.9 (7.69%)	799.8 (21.9%)	27.4 (6.19%)
Intra-batch CV		0.279 (26.45%)	0.580 (8.74%)	83.9 (27.28%)	1212.1 (37.58%)	26.5 (5.51%)
3	1	0.545 (2.98%)	0.408 (2.09%)	131.8 (3.00%)	1803.3 (5.35%)	28.6 (3.35%)
	2	0.242 (4.26%)	0.563 (1.19%)	67.3 (11.73%)	807.3 (13.44%)	28.6 (1.31%)
Intra-batch CV		0.393 (41.19%)	0.485 (17.19%)	99.5 (35.10%)	1305.3 (41.43%)	28.6 (2.36%)
Inter-batch CV		0.329 (34.17%)	0.533 (12.62%)	79.0 (41.18%)	1165.1 (42.28%)	27.28 (6.41%)

**Table 3:** The 90% confidence intervals of the rheological parameters of three batches of 0.025% *w*/*w* capsaicin emulsions: viscosity (η), relative thixotropic loop area (RTLA), complex modulus (G*), and phase angle of delta tangent (δ).

	η 300 s^−1^ (Pa)	RTLA	Yield Stress (Pa)	G* (Pa)	δ (°)
1 vs. 2	1.04–1.21	0.89–0.94	0.53–0.75	0.65–0.97	0.98–1.04
1 vs. 3	0.68–0.92	1.05–1.14	0.42–0.66	0.59–0.91	0.91–0.96
2 vs. 3	0.58–0.83	1.14–1.25	0.72–0.96	0.77–1.08	0.90–0.95

**Table 4:** Intermediate precision of the IVRT study. Table shows mean value, and values in brackets show the relative standard deviation (CV%) for the released amount of capsaicin at 6 h (Q_6h_) and release constant (K).

	Q_6h_ (µg/cm^2^)	K (µg/h^1/2^/cm^2^)
Operator 1	62.8 ± 5.8 (9.25%)	31.7 ± 2.7 (8.65%)
Operator 2	63.9 ± 6.00 (9.39%)	31.9 ± 2.60 (8.14%)
Mean	63.8	31.8
SD	5.8	2.6
CV (%)	9.2	8.1

**Table 5:** The 90% confidence intervals (CIs) of release parameters (released amount of capsaicin at 6 h (Q_6h_) and release constant (K)) of a reference formulation versus a reduced viscosity formulation. SD: standard deviation.

Batch	Parameter	Mean	SD	90% CI
Standard batch	Q_6h_ (µg/cm^2^)	60.13	3.48	1.14–1.22
Reduced viscosity batch	Q_6h_ (µg/cm^2^)	70.91	2.92	
Standard batch	K (µg/h^1/2^/cm^2^)	29.99	1.17	1.19–1.28
Reduced viscosity batch	K (µg/h^1/2^/cm^2^)	36.90	2.23	

**Table 6:** Release parameters (released amount of capsaicin at 6 h (Q_6h_) and release constant (K)) values after the modification of operational parameters. SD: standard deviation.

Factor	Parameter	Mean	SD	*p*-Value
Mixing rate	Q_6h_ (µg/cm^2^)	56.86	8.50	0.250
	K (µg/h^1/2^/cm^2^)	28.93	3.02	0.057
Placed amount of product	Q_6h_ (µg/cm^2^)	57.36	2.33	0.294
	K (µg/h^1/2^/cm^2^)	29.76	1.75	0.132
Temperature of receptor medium	Q_6h_ (µg/cm^2^)	37.64	2.12	0.000
	K (µg/h^1/2^/cm^2^)	18.83	1.41	0.000
Receptor medium compossition	Q_6h_ (µg/cm^2^)	57.12	5.51	0.272
	K (µg/h^1/2^/cm^2^)	29.73	1.39	0.129

**Table 7:** The 90% confidence intervals (CIs) of the release parameters (released amount of capsaicin at 6 h (Q_6h_) and release constant (K)) of three 0.025% *w*/*w* capsaicin emulsion batches. SD: standard deviation.

Batches	*n*	Q_6h_ (µg/cm^2^)				K (µg/h^1/2^/cm^2^)			
		Mean	SD	Ratio	CI (90%)	Mean	SD	Ratio	CI (90%)
1	12	57.27	4.08	1.010	0.928–1.105	28.69	2.23	1.021	0.922–1.139
2	12	56.69	8.88			28.11	5.38		
3	12	56.77	7.36	1.001	0.905–1.108	28.02	4.79	0.997	0.877–1.133
2	12	56.69	8.88			28.11	5.38		
3	12	56.77	7.36	0.991	0.919–1.066	28.02	4.79	0.977	0.885–1.071
1	12	57.27	4.08			28.69	2.23		

**Table 8:** The 90% confidence intervals (CIs) of permeation parameters (released amount of capsaicin at 24 h (Q_24h_) and transdermal flux) of batches one and three of capsaicin emulsion at 0.025% *w*/*w* and the negative control (0.05% *w*/*w*). SD: standard deviation.

Batches	*n*	Q_24h_ (µg/cm^2^)				Transdermal Flux (ng/h/cm^2^)			
		Geometric Mean	SD	Ratio	CI (90%)	Geometric Mean	SD	Ratio	CI (90%)
1	6	342.32	198.14	91.80%	0.6698–1.2581	15.08	8.64	90.23%	0.6547–1.2435
3	6	314.25	152.88			13.61	6.89		
1	6	342.32	198.14	67.44%	0.5006–0.9085	15.08	8.64	66.89%	0.4942–0.9054
NC	6	507.60	206.15			22.55	9.13		

## Data Availability

Data are available upon request owing to intellectual property law.
